# Development of a nomogram to predict survival in advanced biliary tract cancer

**DOI:** 10.1038/s41598-023-48889-6

**Published:** 2023-12-06

**Authors:** Hiroshi Imaoka, Masafumi Ikeda, Shogo Nomura, Chigusa Morizane, Takuji Okusaka, Masato Ozaka, Satoshi Shimizu, Kentaro Yamazaki, Naohiro Okano, Kazuya Sugimori, Hirofumi Shirakawa, Nobumasa Mizuno, Sohei Satoi, Hironori Yamaguchi, Rie Sugimoto, Kunihito Gotoh, Keji Sano, Akinori Asagi, Kazuyoshi Nakamura, Makoto Ueno

**Affiliations:** 1 Department of Hepatobiliary and Pancreatic Oncology, National Cancer Center Hospital East, 6-5-1 Kashiwanoha, Kashiwa, Chiba 277-8577 Japan; 2 Japan Clinical Oncology Group Data Center, Clinical Research Support Office, National Cancer Center Hospital, Tokyo, Japan; 3 Hepatobiliary and Pancreatic Oncology, National Cancer Center Hospital, Tokyo, Japan; 4 Hepato-Biliary-Pancreatic Medicine Department, Cancer Institute Hospital of Japanese Foundation for Cancer Research, Tokyo, Japan; 5 Department of Gastroenterology, Saitama Cancer Center, Saitama, Japan; 6 Division of Gastrointestinal Oncology, Shizuoka Cancer Center, Shizuoka, Japan; 7 Department of Medical Oncology, Faculty of Medicine, Kyorin University, Tokyo, Japan; 8 Gastroenterological Center, Yokohama City University Medical Center, Yokohama, Japan; 9 Department of Medical Oncology, Tochigi Cancer Center, Utsunomiya, Japan; 10 Department of Gastroenterology, Aichi Cancer Center Hospital, Nagoya, Japan; 11 Division of Pancreatobiliary Surgery, Department of Surgery, Kansai Medical University, Hirakata, Japan; 12 Department of Clinical Oncology, Jichi Medical University, Shimotsuke, Japan; 13 Department of Hepato-Biliary-Pancreatology, National Hospital Organization Kyushu Cancer Center, Fukuoka, Japan; 14 Department of Surgery, National Hospital Organization Osaka National Hospital, Osaka, Japan; 15 Department of Surgery, Teikyo University School of Medicine, Tokyo, Japan; 16 Department of Gastrointestinal Medical Oncology, National Hospital Organization Shikoku Cancer Center, Matsuyama, Japan; 17 Division of Gastroenterology, Chiba Cancer Center, Chiba, Japan; 18 Department of Gastroenterology, Hepatobiliary and Pancreatic Medical Oncology Division, Kanagawa Cancer Center, Yokohama, Japan

**Keywords:** Chemotherapy, Biliary tract cancer

## Abstract

The prognosis of advanced biliary tract cancer (BTC) patients remains poor due to limited efficacy of chemotherapy and difficulties in management. Thus, prediction of survival is crucial for the clinical management of advanced BTC. The aim was to develop and validate a nomogram to predict 6-month and 12-month survival in advanced BTC patients treated with chemotherapy. A multivariable Cox regression model was used to construct a nomogram in a training set (JCOG1113, a phase III trial comparing gemcitabine plus S-1 [GS] and gemcitabine plus cisplatin, n = 351). External validity of the nomogram was assessed using a test set (JCOG0805, a randomized, phase II trial comparing GS and S-1 alone, n = 100). Predictive performance was assessed in terms of discrimination and calibration. The constructed nomogram included lymph node metastasis, liver metastasis, carbohydrate antigen 19-9, carcinoembryonic antigen, albumin, and C-reactive protein. Uno’s concordance index was 0.661 (95% confidence interval [CI] 0.629–0.696) in the training set and 0.640 (95% CI 0.566–0.715) in the test set. The calibration plots for 6-month and 12-month survival showed good agreement in the two analysis sets. The present nomogram can facilitate prediction of the prognosis of advanced BTC patients treated with chemotherapy and help clinicians’ prognosis-based decision-making.

## Introduction

Biliary tract cancer (BTC), including intrahepatic cholangiocarcinoma, extrahepatic cholangiocarcinoma, gallbladder cancer, and ampullary cancer, is relatively rare worldwide. However, it is estimated that, in 2017, approximately 210,000 new patients were diagnosed with this disease worldwide^1,2^. Despite differences in anatomical sites and geographical regions, the incidence of BTC has increased^1,3^, and one epidemiological study reported that it increased by 76% between 1990 and 2017^1^. Moreover, the majority of the patients presented in an advanced stage at the time of diagnosis, and such patients are basically treated with chemotherapy^4^. Since the gemcitabine plus cisplatin (GC) regimen was shown to prolong survival in patients with advanced BTC^5^, various chemotherapeutic regimens have been developed. Above all, gemcitabine, platinum, and fluorouracil are widely used as key drugs for advanced BTC^6–11^. However, the patients’ prognosis remains poor, with a median overall survival (OS) of about 1 year^3^.

One possible reason for the poor prognosis is the difficulty in the management of BTC. Differences in anatomical site, clinical presentation, comorbidity, and surgical therapy lead to further challenges in treatment for BTC for many physicians^4,12^. In such situations, prediction of survival is crucial for the clinical management of advanced BTC. However, there are few reports of prognostic models for advanced BTC^13^, and the majority of these studies were single-center, retrospective designs^14–16^. The major drawback of these studies was that the number of censored subjects accounted for 25% to 50%^15,17,18^, which could attenuate the predictability of the built prognostic models for the external datasets. In fact, despite the heterogeneity of BTC, the external validity of these models has not been fully evaluated^19^. Therefore, to develop the prognostic model for advanced BTC, it is necessary to construct a model based on prospectively collected, high-quality data and evaluate the validity of the model. The Japan Clinical Oncology Group (JCOG) previously reported the results of two multicenter, randomized trials that evaluated gemcitabine, cisplatin, and the oral fluorouracil agent S-1. The first one was JCOG0805 (a randomized phase II trial, UMIN Clinical Trials Registry [http://www.umin.ac.jp/ctr/index.htm] number, UMIN000001685), comparing gemcitabine plus S-1 (GS) and S-1 alone, which reported more promising efficacy of GS in the 1-year OS^20^. The second one was JCOG1113 (a phase III trial after JCOG0805, UMIN Clinical Trials Registry number, UMIN000010667), comparing GS and GC in patients with advanced BTC, which demonstrated non-inferiority of GS to GC in OS, but superiority was not shown^9^. Consequently, the GS regimen, as well as the GC regimen, was positioned as one of the standard treatments for advanced BTC in Japan.

Using the two trials’ datasets, an ancillary analysis (termed JCOG1917A) was conducted to develop a nomogram to predict 6-month and 12-month survival proportions.

## Results

### Patients’ characteristics

**Figure 1:**
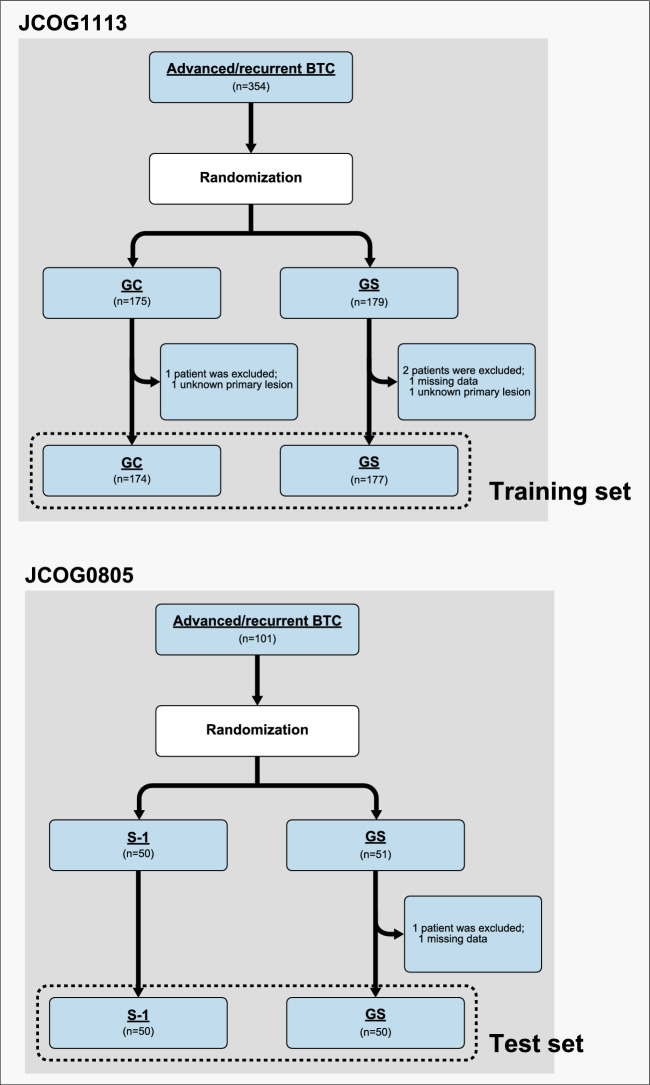
Patient flow chart. *BTC* biliary tract cancer, *GC* gemcitabine plus cisplatin, *GS* gemcitabine plus S-1.

The patient flow chart is shown in Fig. 1. The training set consisted of 351 advanced BTC patients who were enrolled in JCOG1113 between May 2013 and March 2016 (174 in arm GC, and 177 in arm GS). The test set consisted of 100 advanced BTC patients who were enrolled in JCOG0805 between February 2009 and April 2010 (50 in arm GS, and 50 in arm S-1) (Fig. 1). Their baseline characteristics are summarized in Table 1.

**Table 1:** Patients’ characteristics of the training and test sets.

	Training set (n = 351)	Test set (n = 100)
Age (years)		
Median (interquartile range)	67.0 (62.0–73.0)	64.5 (59.0–72.0)
Sex		
Male (%)	195 (55.6)	55 (55.0)
Female (%)	156 (44.4)	45 (45.0)
ECOG performance status		
0 (%)	251 (71.5)	76 (76.0)
1 (%)	100 (28.5)	24 (24.0)
BMI (kg/m^2^)		
Median (interquartile range)	21.7 (19.7–23.6)	21.5 (19.9–23.8)
Primary tumor location		
Intrahepatic bile duct (%)	94 (26.8)	35 (35.0)
Gallbladder (%)	137 (39.0)	37 (37.0)
Extrahepatic bile duct (%)	108 (30.8)	20 (20.0)
Ampulla of Vater (%)	12 (3.4)	8 (8.0)
Extent of disease		
Locally advanced (%)	63 (17.9)	16 (16.0)
Metastatic (%)	288 (82.1)	84 (84.0)
Measurable metastatic sites		
Lymph node (%)	185 (52.7)	64 (64.0)
Liver (%)	130 (37.0)	45 (45.0)
Lung (%)	38 (10.8)	14 (14.0)
Unresectability due to extensive biliary involvement		
Present (%)	26 (7.4)	8 (8.0)
Unresectability due to vascular invasion		
Present (%)	79 (22.5)	24 (24.0)
CA19-9 (U/mL)		
Median (interquartile range)	171.0 (25.6–1746.4)	380.4 (31.8–2976.8)
CEA (ng/mL)		
Median (interquartile range)	3.8 (2.2–12.7)	5.4 (2.4–13.7)
Albumin (g/dL)		
Median (interquartile range)	3.8 (3.4–4.1)	3.8 (3.4–4.0)
CRP (mg/dL)		
Median (interquartile range)	0.50 (0.17–1.59)	0.75 (0.25–1.96)
Neutrophils (/µL)		
Median (interquartile range)	3795.0 (2850.5–5142.5)	4349.0 (3140.0–5930.2)
Alkaline phosphatase (IU/L)		
Median (interquartile range)	452.0 (288.5–733.0)	438.5 (280.5–866.8)

*ECOG PS* Eastern Cooperative Oncology Group performance status, *BMI* body mass index, *CA19-9* carbohydrate antigen 19–9, *CEA* carcinoembryonic antigen, *CRP* C-reactive protein.

### Survival

**Figure 2:**
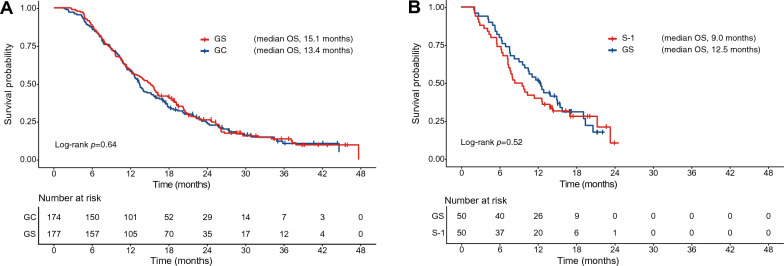
Kaplan–Meier curves comparing overall survival in advanced biliary tract cancer (BTC) patients treated with GC and GS in the training set (**A**), and in advanced BTC patients treated with GS and S-1 in the test set (**B**). *GC* gemcitabine plus cisplatin, *GS* gemcitabine plus S-1.

The median follow-up was 13.8 months in the training set and 10.5 months in the test set. The Kaplan–Meier curves of OS are shown in Fig. 2A,B. In the training set, the 6-month and 12-month OS for all patients were 87.5% and 58.7%, respectively (GS arm: 6-month OS, 88.7%; 12-month OS, 59.3%; GC arm: 6-month OS, 86.2%; 12-month OS, 58.0%). In the test set, the 6-month and 12-month OS were 77.0% and 46.0%, respectively (GS arm: 6-month OS, 80.0%; 12-month OS, 52.0%; S-1 arm: 6-month OS, 74.0%; 12-month OS, 40.0%).

### Development and validation of the nomogram

**Figure 3:**
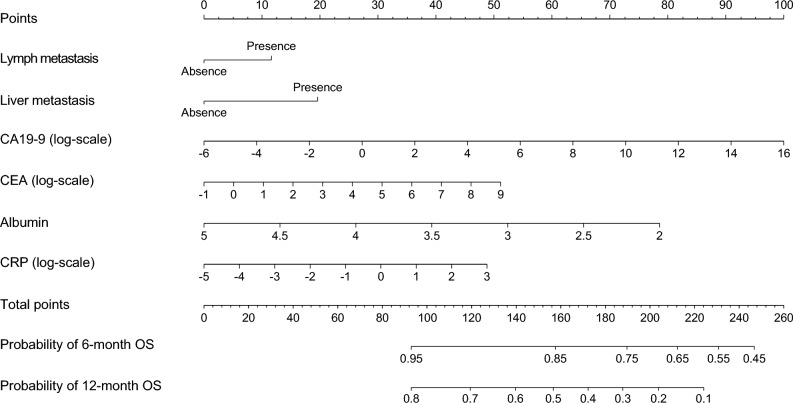
Nomogram for predicting 6-month and 12-month survival in patients with biliary tract cancer. To use the nomogram, an individual patient’s value is located on each variable axis, and a line is drawn upward to determine the number of points received for each variable value. The sum of these numbers is located on the Total Points axis, and a line is drawn downward to the survival axes to determine the probability of 6-month or 12-month survival.

The backward elimination procedure in the Cox regression analysis identified 6 factors (lymph node metastasis, liver metastasis, carbohydrate antigen 19-9 [CA19-9; log scale], carcinoembryonic antigen [CEA; log scale], albumin, and C-reactive protein [CRP; log scale]) in the training set. The final model is summarized in the left panel of Table 2, and the nomogram for 6-month and 12-month survivals is shown in Fig. 3.

**Table 2:** Results of the multivariable Cox models, including the nomogram variables for overall survival.

	HR	Training set	*P*-value	HR	Test set	*P*-value
		95% CI			95% CI	
Lymph node metastasis						
Present vs absent	1.232	0.971–1.563	0.086	1.612	0.946–2.748	0.079
Liver metastasis						
Present vs absent	1.420	1.115–1.808	0.004	2.023	1.232–3.322	0.005
CA19-9 (log-scale)						
Continuous, U/mL	1.084	1.038–1.133	< 0.001	1.037	0.956–1.126	0.381
CEA (log-scale)						
Continuous, ng/mL	1.096	1.016–1.181	0.018	1.131	0.985–1.299	0.081
Albumin						
Continuous, g/dL	0.627	0.477–0.824	0.001	0.771	0.424–1.402	0.394
CRP (log-scale)						
Continuous, mg/dL	1.115	1.012–1.229	0.028	1.257	1.034–1.529	0.022

*HR* hazard ratio, *CI* confidence interval, *CA19-9* carbohydrate antigen 19-9, *CEA* carcinoembryonic antigen, *CRP* C-reactive protein.

**Figure 4:**
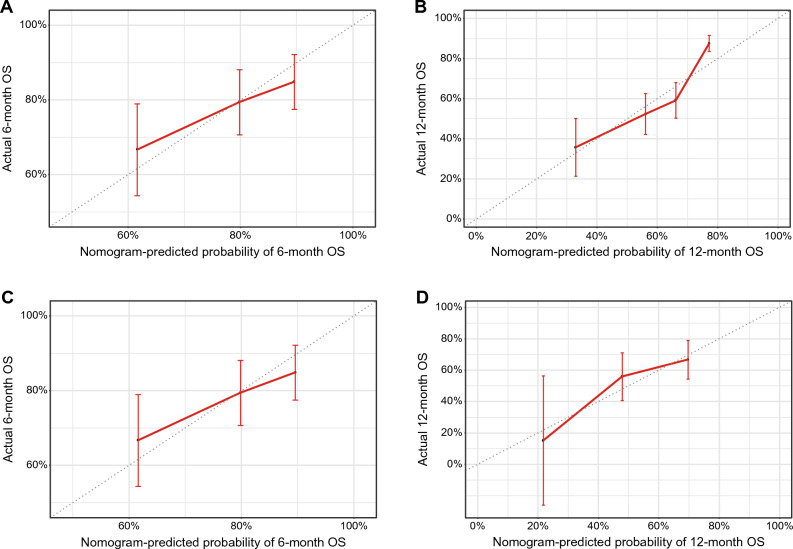
Calibration plots of overall survival probabilities at 6 months (**A**) and 12 months (**B**) in the training set, and at 6 months (**C**) and 12 months (**D**) in the test set. The nomogram-predicted probability of overall survival is plotted on the X-axis, with actual overall survival on the Y-axis. Dashed lines along the diagonal line through the origin point represent the perfect calibration models in which the predicted probabilities are identical to the observed probabilities. *CA19-9* carbohydrate antigen 19-9, *CEA* carcinoembryonic antigen, *CRP* C-reactive protein, *OS* overall survival.

In the training set, the time-dependent receiver-operating characteristic (ROC) is shown in Supplemental Fig. 1, and the AUC was approximately 0.614–0.656 for the thresholds examined. Uno’s concordance index was 0.661 (95% confidence interval [CI] 0.629–0.696). The optimism-corrected value calculated from a bootstrap sample was 0.644. The calibration plots comparing the nomogram-predicted 6-month and 12-month survivals with their corresponding Kaplan–Meier estimates showed good agreement (Fig. 4A,B). In the test set, Uno’s concordance index was 0.640 (95% CI 0.566–0.715), and the optimism-corrected value was 0.640. The calibration plots also showed good agreement (Fig. 4C,D).

### Validation and risk stratification in each treatment arm

**Figure 5:**
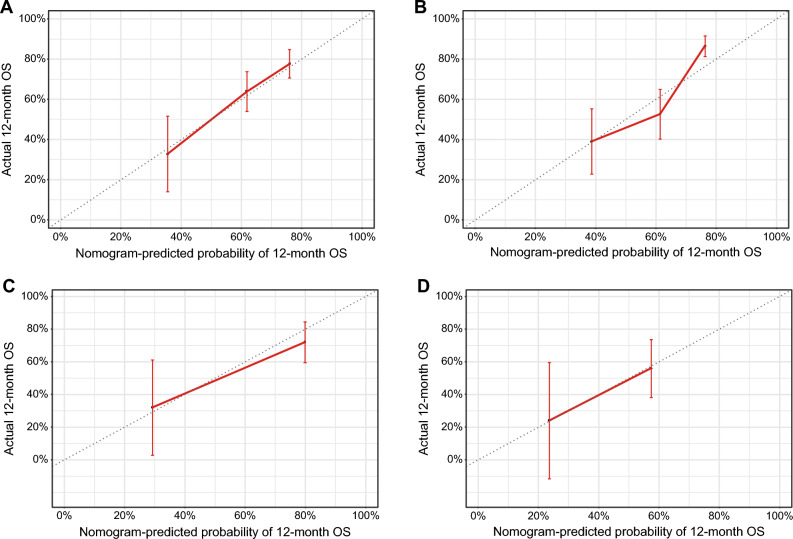
Calibration plots of overall survival probabilities at 12 months in the GC arm of the training set (**A**), the GS arm of the training set (**B**), the GS arm of the test set (**C**), and the S-1 arm of the test set (**D**). *OS* overall survival.

Discrimination and calibration were further assessed in each treatment arm. In the training set, Uno’s concordance indices were 0.662 (GC arm) and 0.661 (GS arm). The indices in the test set were 0.613 (GS arm) and 0.612 (S-1 arm). On the calibration plots by treatment arm for predicting 12-month OS, the calibration plots showed good agreement (Fig. 5A–D). However, slight upper deviation was observed at higher predicted probabilities in the GS arm in the training set.

**Figure 6:**
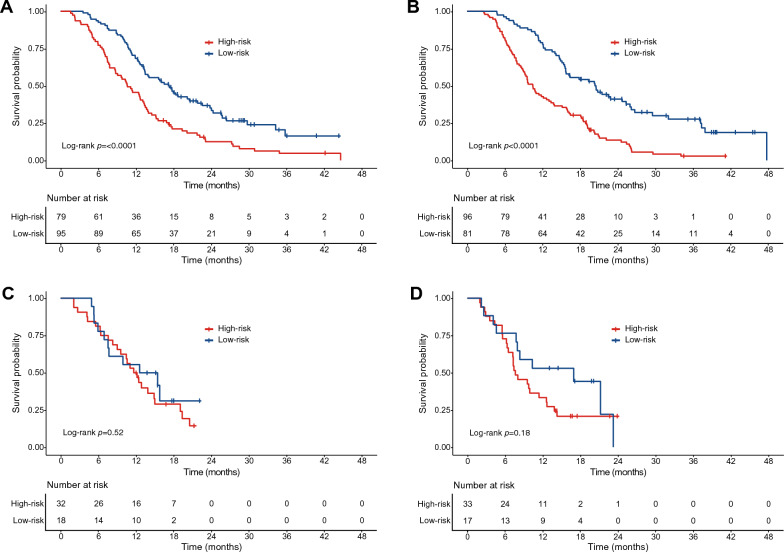
Kaplan–Meier curves comparing overall survival in high-risk and low-risk groups of advanced BTC patients treated with GC in the training set (**A**), in high-risk and low-risk groups of advanced BTC patients treated with GS in the training set (**B**), in high-risk and low-risk groups of advanced BTC patients treated with GS in the test set (**C**), and in high-risk and low-risk groups of advanced BTC patients treated with S-1 in the test set (**D**). *GC* gemcitabine plus cisplatin, *GS* gemcitabine plus S-1.

In addition, patients in the training and test sets were stratified into two groups (high-risk *vs.* low-risk) according to the median value of the nomogram-predicted score. The Kaplan–Meier curves stratified by treatment regimen indicated that the nomogram could discriminate patients into high-risk and low-risk groups in the training set (Fig. 6A,B), but it did so poorly in the test set (Fig. 6C,D).

## Discussion

A nomogram for survival was constructed and validated in patients treated with chemotherapy for advanced BTC using data derived from two randomized trials. The nomogram included lymph node metastasis, liver metastasis, CA19-9, CEA, albumin, and CRP, and its external validity was not problematic in the test set. The use of this nomogram will facilitate prognosis prediction for patients with advanced BTC and help inform patients in the clinical setting. Furthermore, the nomogram also showed calibration abilities in 2 gemcitabine-based chemotherapeutic regimens (GC and GS), as well as S-1 monotherapy. The derived prognostic score based on the nomogram modestly stratified patients into high-risk and low-risk groups in these different regimens for advanced BTC, and these findings suggest that the nomogram can potentially be generalized to other cytotoxic chemotherapeutic regimens.

One strength of the nomogram is its simplicity. A nomogram is a simple-to-use graphical tool and intuitive representation of the statistical model. In recent years, it has been applied in the medical field and used for personalized assessment of risk in patients with various types of cancer^21^. The present nomogram contains 6 variables (lymph nodes metastasis, liver metastasis, CA19-9, CEA, albumin, and CRP), all of which are commonly used in daily medical practice. Thus, this nomogram can be easily applied in daily clinical use. Other strengths of this study include external validation of the findings and use of prospectively collected clinical trial data. The American Joint Committee on Cancer (AJCC)/TNM staging system is the most widely used classification for prognostic estimates, but the prognostic performance of AJCC/TNM staging has mainly been validated in surgical cases and rarely in unresectable cases^22^. Therefore, the AJCC/TNM staging system may not be suitable for risk stratification of patients with unresectable disease. A previous study by Wu et al. developed a nomogram for advanced BTC patients treated with gemcitabine-based chemotherapy using retrospectively collected data^14^. Their nomogram-predicted 6-month survival outcomes were well correlated with the actual observed ones in the analysis set for model construction. Generally, the median OS of advanced BTC patients treated with gemcitabine-based chemotherapy reaches about 1 year, and the long-term predictability of their proposed nomogram was uncertain. The present nomogram was developed using prospectively collected data from multicenter clinical trials and validated externally. Therefore, the results should be more applicable to the general population than models whose external validity has not been assessed. However, the credibility and validity of this nomogram for patients who do not meet the inclusion criteria or who meet the exclusion criteria for the two trials have not been established.

The factors constituting the present nomogram are categorized mainly into two groups: tumor-related factors and inflammatory-related factors. Tumor-related factors, lymph node metastasis, liver metastasis, CA19-9, and CEA, represent tumor burden, and their importance as prognostic factors is widely accepted^16,23–26^. On the other hand, the inflammatory-related factors have recently been highlighted. Cancer-related inflammation leads to alterations of the tumor microenvironment and contributes to cancer cell proliferation, invasion, and metastatic spread^27^. Furthermore, this inflammation also affects cancer development by increasing catabolism and impairing nutrient absorption. Conversely, malnutrition promotes the exacerbation of inflammation^28^. Thus, inflammation and malnutrition are interrelated and promote cancer progression. Albumin and CRP are well-known inflammation-related prognostic factors, and their prognostic values have been confirmed in a variety of solid tumors^29^. However, their utilities for advanced BTC have been not well examined up to now. In the present study, the addition of inflammatory-related factors strengthened the prognostic capabilities of the nomogram, and this fact indicates that systemic inflammation plays a major prognostic role in advanced BTC. Furthermore, it is noteworthy that our model comprehensively predicts the prognosis of BTC from both tumor and inflammation aspects compared to previous reports^18,30,31^.

The present nomogram not only enables simple application to predict survival, but it also helps clinicians’ prognosis-based decision-making. If poor outcomes are predicted by the nomogram, early comprehensive genomic profiling is recommended for precision medicine. After first-line gemcitabine-based chemotherapy, fluorouracil-based chemotherapy is commonly used for advanced BTC, but its efficacy is modest^32^. However, novel biomarker-based therapies^33–35^, such as isocitrate dehydrogenase 1 (IDH1) inhibitors for *IDH1* mutations^36^, fibroblast growth factor receptor (FGFR) inhibitors for *FGFR2* fusion/rearrangement^37–41^, and immune checkpoint inhibitors (ICIs) for microsatellite instability-high/mismatch repair-deficiency^42^, have been emerging for advanced BTC, and they have shown promising results in these patients with a poor prognosis. Thus, early genomic profiling opens up the possibility of new treatments for patients who are expected to have a poor prognosis with standard treatment. Prognostic prediction by the present nomogram may accelerate the application of precision medicine and contribute to prognosis-based decision-making in the therapeutic strategy for advanced BTC patients.

The present study had some limitations. The first limitation is the fact that the nomogram was developed using integrated data with limited sample size. However, the predictive performance of the present nomogram was good in the validation set. The second limitation is that this nomogram was developed based on data of patients treated only with cytotoxic chemotherapy. Recently, a phase III trial showed that a combination of ICI and cytotoxic chemotherapy (durvalumab plus GC) improved OS compared with cytotoxic chemotherapy alone (GC)^11^. It is unclear whether the present nomogram can be used for patients treated with ICIs. The third limitation is that the discriminative ability of the nomogram was relatively modest. This could be due to the heterogeneity exhibited by BTCs. BTCs are subclassified into important subtypes, such as intrahepatic cholangiocarcinoma and gallbladder cancer, each with its own unique characteristics including prognosis. The test set in this study had a higher proportion of intrahepatic cholangiocarcinoma than the train set. This fact may contribute to the relatively modest discriminative ability. The fourth limitation is that, due to the sample size, our model can only classify patients into two risk groups: high-risk and low-risk. Previous reports have classified patients into three groups: high-risk, intermediate-risk, and low-risk^18^, which may be more appropriate for practical use.

In conclusion, a nomogram for predicting survival in patients treated with chemotherapy for advanced BTC was developed using data derived from two randomized trials. The nomogram successfully demonstrated external validity and also showed calibration abilities for three different chemotherapeutic regimens. This nomogram will facilitate prognosis prediction for patients with advanced BTC, help clinicians’ prognosis-based decision-making, and inform patients in the clinical setting.

## Methods

A total of 455 patients were enrolled in JCOG0805 (101 patients) and JCOG1113 (354 patients). Details of the study design, inclusion/exclusion criteria, and efficacy and safety results have been described previously^9,20^. Briefly, the primary endpoint of JCOG1113 was OS, and that of JCOG0805 was 1-year OS. The protocol of this ancillary study (JCOG1917A) was approved by the institutional review board of National Cancer Center Hospital East. Written informed consent, including secondary use of data, was obtained from each patient before enrollment in JCOG1113 or JCOG0805. This study was performed in accordance with the international ethical recommendations of the Declaration of Helsinki and the Ethical Guidelines for Medical and Health Research Involving Human Subjects.

Of all 455 randomized patients, four were excluded, because two patients in JCOG1113 had indistinguishable primary sites and the other two patients (one in JCOG1113 and one in JCOG0805) had missing data for factors needed to construct the nomogram. Training and test sets consisted of 351 and 100 patients, respectively.

The endpoint of interest was OS, defined as the duration between the date of randomization and the date of death or censored on the last date they were known to be alive. In the nomogram, prediction performance of survival proportions at 6 and 12 months was also evaluated, since the median OS in first-line chemotherapy for advanced BTC is about 1 year^5,9,11^.

### Statistical considerations

Multivariable Cox regression analyses were performed to assess the associations between various factors and OS. The factors included were age (continuous)^17,43,44^, sex (male or female)^3,45,46^, Eastern Cooperative Oncology Group (ECOG) performance status (0 or 1)^15,16,23^, body mass index (BMI; continuous)^47,48^, primary tumor location (intrahepatic bile duct, gallbladder, extrahepatic bile duct, or ampulla of Vater)^3,49^, extent of disease (locally advanced or metastatic), lymph node metastasis (present or absent)^50^, liver metastasis (present or absent)^16^, lung metastasis (present or absent), unresectability due to extensive biliary involvement (yes or no)^51^, unresectability due to vascular invasion (yes or no)^50,51^, CA19-9 (continuous)^24^, CEA (continuous)^23^, albumin (continuous)^52^, CRP (continuous)^24,52^, neutrophils (continuous)^13,15^, and alkaline phosphatase (continuous)^16^. Logarithmic transformations were performed for continuous variables, except age, BMI, and albumin, because their empirical distributions were highly skewed with a heavy tail. For CA19-9 and CRP, a small value of 0.01 was added to handle the logarithmic transformation of zero. A non-linear association of age and neutrophils (logarithmic scale) with the log hazard ratio was observed on univariable Cox regression analysis, and thus the two factors were modeled as restricted cubic spline functions with 4 knots. The final model was selected following a backward elimination procedure using Akaike’s information criterion as a stopping rule. The nomogram for 6-month and 12-month OS was formulated with rms package version 6.2 in R.

Predictive performance of the constructed nomogram was assessed in two ways (discrimination and calibration) with the training set^53^. Discrimination was first graphically assessed using time-dependent ROC curves for every six months until 48 months^54^. Uno’s concordance index was calculated, and its optimistic-corrected value was estimated by bootstrapping with 1000 resamples^55^. Calibration was next evaluated by comparing nomogram-predicted survival with their Kaplan–Meier estimates in 4 groups classified based on a linear predictor of the final model. Calibration was assessed by plotting the predicted values against the actual estimates, which should be distributed near a 45-degree line for a well-calibrated model.

Finally, external validation was performed with the test set in the same manner as with the training set. Considering the limited sample size, calibration was done in 3 groups classified based on a linear predictor. Calibration was further assessed by stratifying by treatment arm in the training and test sets. All analyses were performed with R version 4.1.2. All reported P-values are two-sided.

### Supplementary Information

Supplementary Figure 1.

## Data Availability

The data underlying this article cannot be shared publicly due to protection of privacy of individuals who participated in the study. The data will be shared with investigators whose proposed use of the data has been approved by investigators from the JCOG Hepatobiliary and Pancreatic Oncology Group. Proposals should be directed to the corresponding author.
