# Supplementary material for: Development of a nomogram to predict survival in advanced biliary tract cancer
Source: Sci Rep. 2023 Dec 6;13:21548. doi: 10.1038/s41598-023-48889-6 (PMC10700490; doi:10.1038/s41598-023-48889-6)
Supplement: Supplementary file 1 — Supplementary Figure 1. [file 41598_2023_48889_MOESM1_ESM.pdf]

Development of a nomogram to predict survival in advanced biliary tract cancer

Hiroshi Imaoka[1], Masafumi Ikeda[1], Shogo Nomura[2], Chigusa Morizane[3], Takuji Okusaka[3], Masato Ozaka[4], Satoshi Shimizu[5], Kentaro Yamazaki[6], Naohiro Okano[7], Kazuya Sugimori[8], Hirofumi Shirakawa[9], Nobumasa Mizuno[10], Sohei Satoi[11], Hironori Yamaguchi[12], Rie Sugimoto[13], Kunihito Gotoh[14], Keji Sano[15], Akinori Asagi[16], Kazuyoshi Nakamura[17], Makoto Ueno[18] on behalf of the JCOG Hepatobiliary and Pancreatic Oncology Group.

[1] Department of Hepatobiliary and Pancreatic Oncology, National Cancer Center Hospital East, Kashiwa, Japan

[2] Japan Clinical Oncology Group Data Center, Clinical Research Support Office, National Cancer Center Hospital, Tokyo, Japan

[3] Hepatobiliary and Pancreatic Oncology, National Cancer Center Hospital, Tokyo, Japan

[4] Hepato-Biliary-Pancreatic Medicine Department, Cancer Institute Hospital of Japanese Foundation for Cancer Research, Tokyo, Japan

[5] Department of Gastroenterology, Saitama Cancer Center, Saitama, Japan

[6] Division of Gastrointestinal Oncology, Shizuoka Cancer Center, Shizuoka, Japan

[7] Department of Medical Oncology, Kyorin University Faculty of Medicine, Tokyo, Japan

[8] Gastroenterological Center, Yokohama City University Medical Center, Yokohama, Japan

[9] Department of Medical Oncology, Tochigi Cancer Center, Utsunomiya, Japan

[10] Department of Gastroenterology, Aichi Cancer Center Hospital, Nagoya, Japan

[11] Division of Pancreatobiliary Surgery, Department of Surgery, Kansai Medical University, Hirakata, Japan

[12] Department of Clinical Oncology, Jichi Medical University, Shimotsuke, Japan

[13] Department of Hepato-Biliary-Pancreatology, National Hospital Organization Kyushu Cancer Center, Fukuoka, Japan

[14] Department of Surgery, National Hospital Organization Osaka National Hospital, Osaka, Japan
[15] Department of Surgery, Teikyo University School of Medicine, Tokyo, Japan
[16] Department of Gastrointestinal Medical Oncology, National Hospital Organization Shikoku Cancer Center, Matsuyama, Japan
[17] Division of Gastroenterology, Chiba Cancer Center, Chiba, Japan
[18] Department of Gastroenterology, Hepatobiliary and Pancreatic Medical Oncology Division, Kanagawa Cancer Center, Yokohama, Japan

Corresponding Author: Hiroshi Imaoka, M.D., Ph.D.
Department of Hepatobiliary and Pancreatic Oncology, National Cancer Center Hospital East, 6-5-1 Kashiwanoha, Kashiwa, Chiba 277-8577, Japan
Tel: +81-4-7133-1111; Fax: +81-4-7133-0355
E-mail: hiimaoka@east.ncc.go.jp
ORCID: 0000-0003-0584-0095

Supplemental Figure 1

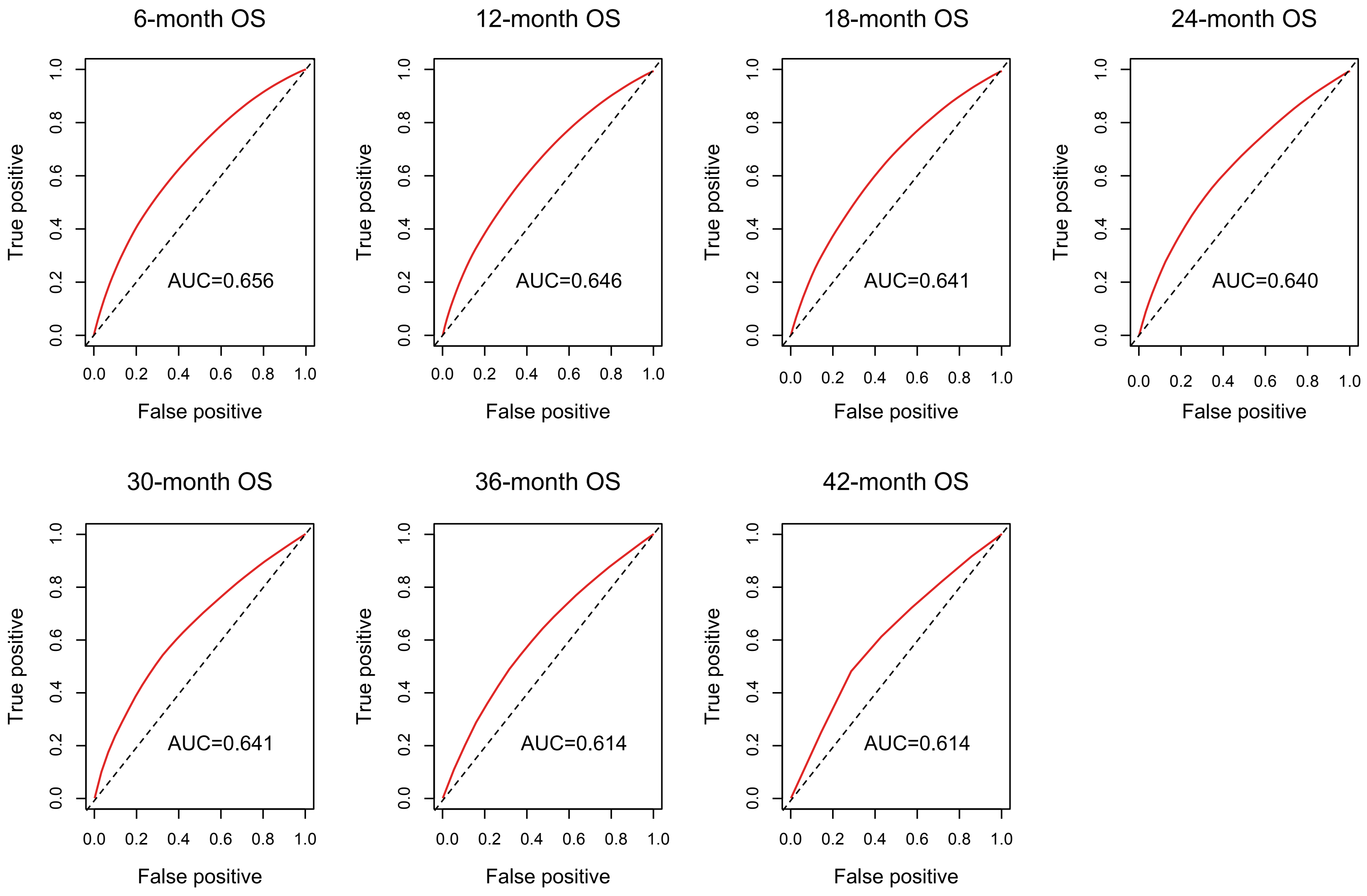

Time-dependent receiver operating characteristic curves for every 6 months until 48 months in the training set. OS, overall survival; AUC, area under the curve.
